# Persistent Systemic Microbial Translocation and Intestinal Damage During Coronavirus Disease-19

**DOI:** 10.3389/fimmu.2021.708149

**Published:** 2021-07-14

**Authors:** Alessandra Oliva, Maria Claudia Miele, Federica Di Timoteo, Massimiliano De Angelis, Vera Mauro, Raissa Aronica, Dania Al Ismail, Giancarlo Ceccarelli, Claudia Pinacchio, Gabriella d’Ettorre, Maria Teresa Mascellino, Claudio M. Mastroianni

**Affiliations:** Department of Public Health and Infectious Diseases, Sapienza University of Rome, Rome, Italy

**Keywords:** COVID-19, SARS-CoV-2, microbial translocation, intestinal damage, lipopolysacharide binding protein, intestinal fatty acid binding protein (I-FABP), microbial translocation in COVID-19, intestinal damage and permeability

## Abstract

Microbial translocation (MT) and intestinal damage (ID) are poorly explored in COVID-19. Aims were to assess whether alteration of gut permeability and cell integrity characterize COVID-19 patients, whether it is more pronounced in severe infections and whether it influences the development of subsequent bloodstream infection (BSI). Furthermore, we looked at the potential predictive role of TM and ID markers on Intensive Care Unit (ICU) admission and in-hospital mortality. Over March–July 2020, 45 COVID-19 patients were enrolled. Markers of MT [LPB (Lipopolysacharide Binding Protein) and EndoCab IgM] and ID [I-FABP (Intestinal Fatty Acid Binding Protein)] were evaluated at COVID-19 diagnosis and after 7 days. As a control group, age- and gender-matched healthy donors (HDs) enrolled during the same study period were included. Median age was 66 (56-71) years. Twenty-one (46.6%) were admitted to ICU and mortality was 22% (10/45). Compared to HD, a high degree of MT and ID was observed. ICU patients had higher levels of MT, but not of ID, than non-ICU ones. Likewise, patients with BSI had lower EndoCab IgM than non-BSI. Interestingly, patients with high degree of MT and low ID were likely to be admitted to ICU (AUC 0.822). Patients with COVID-19 exhibited high level of MT, especially subjects admitted to ICU. COVID-19 is associated with gut permeability.

## Introduction

To date, the novel β-coronavirus Severe Acute Respiratory Syndrome Coronavirus 2 (SARS-CoV-2) causing the ongoing pandemic has infected over 132 million people and resulted in more than 2.8 million deaths worldwide (1, 2). Coronavirus Disease-19 (COVID-19) is characterized by different clinical features, ranging from asymptomatic to severe forms, which involve multiple organs including, amongst others, gut (3).

To this extent, Angiotensin-converting enzyme-2 (ACE-2), the main virus receptors, are expressed not only on the upper and lower respiratory tract, but also on endothelial, intestinal and thyroid cells, thus contributing to explain the systemic nature of the disease (4, 5). Following the virus-receptor binding and its internalization, an especially strong inflammatory response that goes beyond what is required to kill the virus, is mounted, resulting in a high release of cytokines, (the so-called cytokine storm), which is responsible for the widespread inflammation and tissue damage in patients with severe COVID-19 (5).

Recent studies have shown that up to one-third of COVID-19 patients also experience gastrointestinal symptoms such as diarrhea and vomiting along with the most common respiratory symptoms, suggesting a gut involvement during the course of infection (6–8).

Intestinal damage and microbial translocation, the latter defined as the migration of bacteria or their products from gut to the extraintestinal space and eventually to the systemic circulation as a consequence of gut mucosal integrity alteration, have been demonstrated to occur in several infectious and non-infectious conditions (9–11). Likewise, a possible pathogenetic role of gut leakage in favoring the development of bloodstream infection (BSI), especially from enteric microorganisms, has been proposed.

Based on these evidences, it could be hypothesized that microbial translocation (MT) and intestinal damage (ID) (12) may be also present during COVID-19.

Considering that biomarkers of MT and ID are still poorly explored in COVID-19, aims of our study were to assess: i) whether an alteration of gut permeability and cell integrity characterize patients during the course of COVID-19, ii) if present, whether the degree of this alteration is more pronounced in severe infections and iii) whether the presence of MT might have a role in the development of BSI following COVID-19. Furthermore, we investigated the potential predictive role of TM and ID markers on Intensive Care Unit (ICU) admission and in-hospital mortality.

## Methods

### Study Population

Over a period of 6 months (March 2020–July 2020), patients with COVID-19 hospitalized at the Azienda Policlinico Umberto 1, Sapienza University (Rome) were enrolled in this study. Demographic, clinical and radiological data from all participants were recorded in an electronic database.

Patients were further divided into those requiring ICU or not and those developing nosocomial BSI during the course of COVID-19 or not. As a control group, non-hospitalized age- and gender-matched healthy donors (HDs, n=16) with negative SARS-CoV-2 RNA enrolled during the same study period (March-July 2020) were included.

The study protocol was approved by the local Ethics Committee and patients gave written informed consent (ID Prot. 298/2020).

### Definitions

Diagnosis of COVID-19 was made based on suggestive clinical symptoms plus detection of SARS-CoV-2 RNA in nasopharyngeal swab samples by using real time RT-PCR assay (RealStar SARS-CoV2 RT-PCR, Altona Diagnostics). All tests and procedures were performed following the manufacturers’ protocols.

Definition of pneumonia or severe pneumonia was based on the WHO interim guidance and included clinical signs of pneumonia (fever, cough, dyspnoea, fast breathing) with or without signs of severe pneumonia such as respiratory rate > 30 breaths/min, severe respiratory distress, or SpO2 < 90% on room air (13).

Marker of infection severity was considered ICU admission and clinical outcome was in-hospital mortality.

BSI was defined as a nosocomial infection (n-BSI) if it occurred >48h after admission to the hospital (14).

### Markers of Microbial Translocation and Intestinal Damage

For each subject, whole blood samples were collected at the moment of (T0) and after 7 days (T7) from the diagnosis of COVID-19, respectively. Ten milliliters of whole blood were collected by venipuncture in Vacutainer tubes containing ethylene-diamine-tetra-acetic acid (EDTA) (BD Biosciences, San Jose, CA) at each study visit. Plasma was immediately separated by centrifugation at 2000 rpm for 10 minutes and further stored at –80°C until the assays were performed.

Markers of MT [LPB (Lipopolysacharide Binding Protein, RayBiotech); EndoCab IgM, Hycult Biotech] and ID [I-FABP (Intestinal Fatty Acid Binding Protein), R&D Systems] were evaluated using enzyme-linked immunosorbent assays in plasma, according to manufacturers’ instructions.

LBP was expressed as ng/mL (sample diluted 1:1000), EndoCab-IgM as MU/mL (sample diluted 1:50) and I-FABP as pg/mL (sample diluted 1:5). Samples from healthy non-hospitalized subjects were also included and collected once.

### Statistical Analyses

Results were expressed as median (interquartile range [IQR]) and as percentages for continuous and categorical variables, respectively. Categorical variables were compared by using the X2 or Fisher exact test, as appropriate, whereas continuous data were analyzed with the Student *t* test and the nonparametric Mann-Whitney test. Differences between T0 and T7 were evaluated by Wilcoxon test. To determine an appropriate cut-off value for mortality and ICU prediction for each MT and ID marker we used the Youden’s Index. The resulting values were inserted in a simple logistic regression model and respective ROC curves and AUC values (IC95%) for differentiating the different outcomes were calculated. Logistic regression was performed to evaluate the association of different parameters with in-hospital mortality.

Statistical analysis was performed using GraphPad Prism, version 8, for Windows (Grafpad Software MacKiev) and STATA/IC software (StataCorp) version 15, as appropriate.

## Results

### Study Population

A total of 45 patients affected by COVID-19, with a median age of 66 (56–71) years, was included in this study; their demographic and clinical characteristics are reported in **Table 1**. Amongst whole population, 27 (60%) were males and 18 (40%) were females and the median duration of symptoms before SARS-CoV-2 diagnosis was 3.7 (2.7-9.7) days. Although the most common symptoms were fever, dyspnoea and cough [33 (73.3%), 20 (44.4%), 14 (31.1%), respectively], gastrointestinal involvement was observed in 4 subjects (8.8%).

**Table 1:** General characteristics of study population.

Characteristics	Study population (n = 45)
Age, years, median (IQR)	66 (56-71)
Sex (M/F), n	27/18
Duration of symptoms,days, median (IQR)	3.7 (2.7-9.7)
**Blood analyses, median (IQR)**	
White Blood Cells, x10^6^/L	6740 (4920-8610)
Neutrophils, x10^6^/L	4670 (3460-7680)
Lymphocytes, x10^6^/L	820 (600-1210)
Monocytes, x10^6^/L	360 (240-510)
Platelets, x10^9^/L	194 (176-248)
Albumin, g/L	3.6 (3.3-4.2)
D-Dimer, µg/L	670.5 (389.5-2139)
Fibrinogen, mg/dL	555 (398.3-575.5)
PaO2/FiO2 ratio	309 (238.5-373.5)
**Comorbidities, n (%)**	
Smoke	3 (6.6)
Diabetes mellitus	7 (15.5)
Heart failure	4 (8.8)
Vasculopathy	10 (22.2)
Cerebrovascular events	4 (8.8)
Asthma	0 (0)
Chronic Obstructive Pulmonary Disease	4 (8.8)
AIDS	0 (0)
**Symptoms, n (%)**	
Fever	33 (73.3)
Cough	14 (31.1)
Dispnoea	20 (44.4)
Diarrhea	4 (8.8)
Headache	2 (4.4)
Fatigue	2 (4.4)
**Therapy, n (%)**	
Hydroxychloroquine	33 (73.3)
Azithromycin	19 (42.2)
Protease inhibitors	7 (15.5)
Tocilizumab	21 (46.6)
Steroids	15 (33.3)
Enoxaparin	24 (53.3)
Teicoplanin	9 (20)
**Outcomes, n (%)**	
Intensive Care Unit admission	21 (46.6)
Trombotic events	6 (13.3)
Bloodstream infection	9 (20)
Death	10 (22.2)

AIDS, Acquired Immune Deficiency Syndrome.

Hydroxychloroquine (HCQ) and antivirals were used in 33/45 (73.3%) and 7/45 (15.5%) subjects, whereas azithromycin and teicoplanin (15–17) were used in 19/45 (42.2%) and 9/45 (20%) subjects, respectively. As adjunctive therapy, tocilizumab, steroids and enoxaparine were administered in 21/45 (46.6%), 15/45 (33.3%) and 24/45 (53.3%) subjects, respectively. Twenty-one patients (46.6%) were admitted to ICU and overall mortality was 22.2% (10/45). Amongst subjects with COVID-19, 9/45 (20%) developed n-BSI [9/21 (42.8%) and 0/24 (0%) in ICU and non-ICU subjects, respectively, p=0.0003]. Although Gram-positive microorganisms were the most common isolates causing BSI (n=5, 55.5%, 2 *Staphylococcus aureus*, 1 *Enterococcus faecalis*, 2 coagulase-negative Staphylococci), Gram-negative ones (n=3, 1 *Acinetobacter baumannii*, 1 *Klebsiella pneumoniae*, 1 *Escherichia coli*) and *Candida* species (n=1) accounted for the remaining 45.5%.

### Markers of Microbial Translocation and Intestinal Damage During the Course of COVID-19

**Figure 1:**
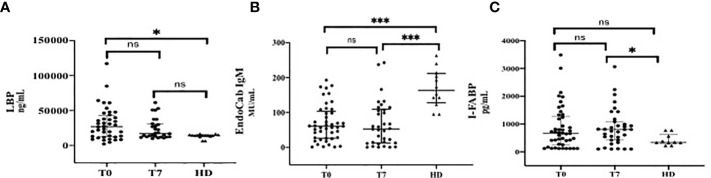
Markers of Microbial Translocation and Intestinal Damage during the Course of COVID-19 in comparison with healthy donors (HD). **(A)** LPB (Lipopolysacharide Binding Protein); **(B)** EndoCab IgM; **(C)** I-FABP (Intestinal Fatty Acid Binding Protein). T0: at the moment of COVID-19 diagnosis; T7: 7 days after COVID-19 diagnosis. ***p < 0.001; *p < 0.05; ns, not significant.

Overall, a higher degree of MT and ID was observed in patients affected by COVID-19 during the course of the disease, compared to HD (**Figure 1**).

In particular, LPB plasma levels (median, IQR ng/ml) at T0 (27030, 12343-42818) were significantly higher than those of HD [14300 (13350-15340), p=0.01]. At T7, a similar trend was observed [17080 (12438-30805), p=0.08] (**Figure 1A**).

The degree of consumption of neutralizing antibodies against LPS endotoxin core antigen, expressed by low levels of EndoCab IgM (median, IQR MU/mL), was significantly higher in COVID-19 patients compared to HD at T0 [60.59 (26.45-103.3) *vs* 163 (128-211.5), respectively, p<0.0001]. This difference was maintained at T7 [53 (12.32-109.1) MU/mL, p< 0.0001] (**Figure 1B**).

Circulating levels of I-FABP (median, IQR pg/mL) at T0 were higher in COVID-19 than those in HD [667.3 (231.7-1266) *vs* 341.6 (302.1-619.4), respectively, p=0.11], although they did not reach statistically significance; by contrast, this difference was remarkable over the course of infection [804.5 pg/mL (422.5-1086), p=0.02] (**Figure 1C**).

Furthermore, we analyzed the abovementioned markers of MT and ID according to the presence (n=4, 8.8%) or absence (n=41, 91.2%) of gastrointestinal symptoms and we found no differences between these groups of subjects (p>0.05 for all these measurements) (*data not shown*).

### Markers of Microbial Translocation and Intestinal Damage During the Course of COVID-19: ICU *Versus* Non-ICU Patients

**Figure 2:**
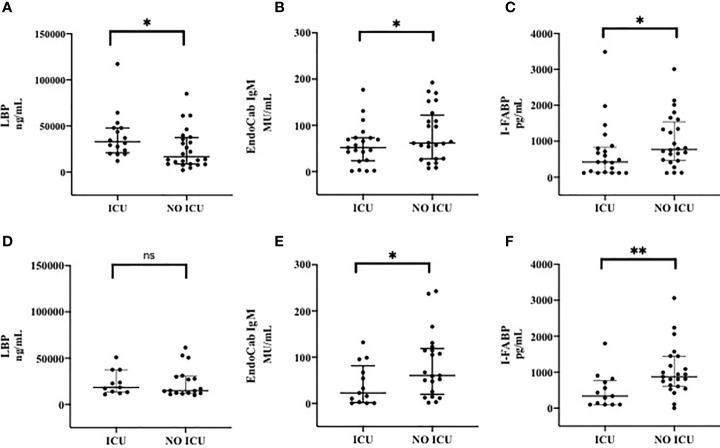
Markers of Microbial Translocation and Intestinal Damage in patients admitted to ICU and patients not admitted to ICU at T0 **(A–C)** and T7 **(D–F)**. **(A)** LPB (Lipopolysacharide Binding Protein) at T0; **(B)** EndoCab IgM at T0; **(C)** I-FABP (Intestinal Fatty Acid Binding Protein) at T0; **(D)** LPB (Lipopolysacharide Binding Protein) at T7; **(E)** EndoCab IgM at T7; **(F)** I-FABP (Intestinal Fatty Acid Binding Protein) at T7. T0: at the moment of COVID-19 diagnosis; T7: 7 days after COVID-19 diagnosis. ICU: Intensive Care Unit. **p < 0.01; *p < 0.5; ns, not significant.

Plasma levels of LBP (median, IQR, ng/mL) at T0 were significantly higher in ICU than those in non-ICU patients [32970 (20935-47835) *vs* 16765 (8748-37390), p= 0.02] (**Figure 2A**), whereas no statistically significant difference was observed at T7 between these two groups (**Figure 2D**).

Moreover, EndoCab-IgM plasma levels (median, IQR, MU/mL) at T0 were lower in patients admitted to ICU compared to non-ICU [51.86 (23.44-73.01) *vs* 61.82 (27.55-122.2), respectively, p=0.2] (**Figure 2B**); additionally, same results in terms of EndoCab-IgM were found to be more evident during the course of disease by comparing the two groups at T7 [22.69 (1.74-81.2) in ICU group *vs* 60.37 (19.77-118.6) in non-ICU group, p=0.05] (**Figure 2E**).

As for I-FABP (median, IQR, pg/mL), statistically significant lower values of this marker were observed at T0 in ICU compared to those recorded in non-ICU patients [422.5 (129.9-835.3) *vs* 772.8 (464.6-1540), respectively, p=0.04] (**Figure 2C**). This difference was even more pronounced during the course of disease by comparing the two groups at T7 [337.3 (100-768.7) in ICU *vs* 873 (606.2-1445) in non-ICU groups, p=0.006] (**Figure 2F**).

Clinical data are depicted in **Supplementary Table 1**.

### Markers of Microbial Translocation and Intestinal Damage During the Course of COVID-19: BSI *vs.* Non-BSI

**Figure 3:**
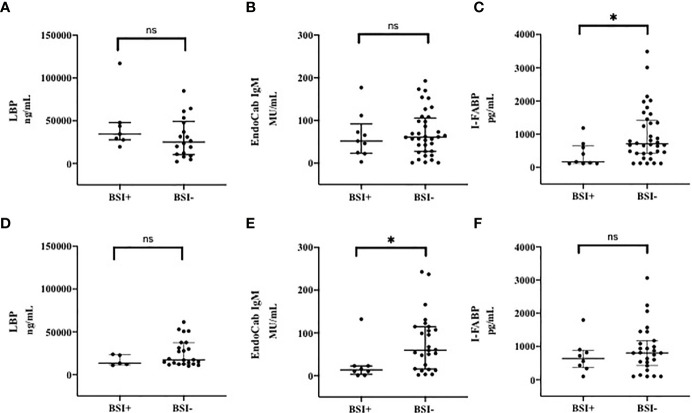
Markers of Microbial Translocation and Intestinal Damage in patients with and without BSI at T0 **(A–C)** and T7 **(D–F)**. **(A)** LPB (Lipopolysacharide Binding Protein) at T0; **(B)** EndoCab IgM at T0; **(C)** I-FABP (Intestinal Fatty Acid Binding Protein) at T0; **(D)** LPB (Lipopolysacharide Binding Protein) at T7; **(E)** EndoCab IgM at T7; **(F)** I-FABP (Intestinal Fatty Acid Binding Protein) at T7. T0: at the moment of COVID-19 diagnosis; T7: 7 days after COVID-19 diagnosis. BSI, nosocomial bloodstream infection. *p < 0.05; ns, not significant.

Following the hypothesis that mucosal perturbation might be responsible for the development of the observed BSI in COVID-19 (18, 19), we also analysed these markers in patients who developed n-BSI and in patients who did not develop n-BSI during hospitalization (**Figure 3**). At T0, patients who developed n-BSI showed a trend toward an increase of LBP (median, IQR, ng/mL) than those without n-BSI [34470 (27580-47790) *vs* 25050 (10334-49250), p=0.24] (**Figure 3A**).

On the other hand, at T7 LBP values (median, IQR, ng/mL) were 13190 (11370-23150) in subjects with n-BSI and 17300 (12460-37470) in those without n-BSI, p=0.21 (**Figure 3D**).

Although not significant at T0, EndoCab IgM levels (median, IQR, MU/mL) were lower (expression of high level of MT) in patients with BSI than in non-BSI [51.86 (23.44-92.18) vs 61.04 (27.55-105.7) p=0.76] (**Figure 3B**), whereas this difference was remarkable at T7 [13.13 (3.03-22.69) *vs* 59.60 (15.55-114.5); p=0.02] (**Figure 3E**).

The level of intestinal damage (I-FABP, median, IQR, pg/mL) was lower in BSI than in non-BSI group at T0 [167.6 (120-655.1) *vs* 708 (422.5-1424), respectively, p=0.01] (**Figure 3C**), but not at T7 (**Figure 3F**). However, during the follow-up period, intestinal damage tended to increase in subjects developing BSI [from 167.6 (120-1184) to 638.9 (360-884.5); p=0.19], whereas in subjects not developing BSI a smaller increase in circulating I-FABP levels was observed.

Clinical data of are depicted in **Supplementary Table 2**.

### Predictive Values of Microbial Translocation and Intestinal Damage Markers on ICU Admission and In-Hospital Mortality

**Figure 4:**
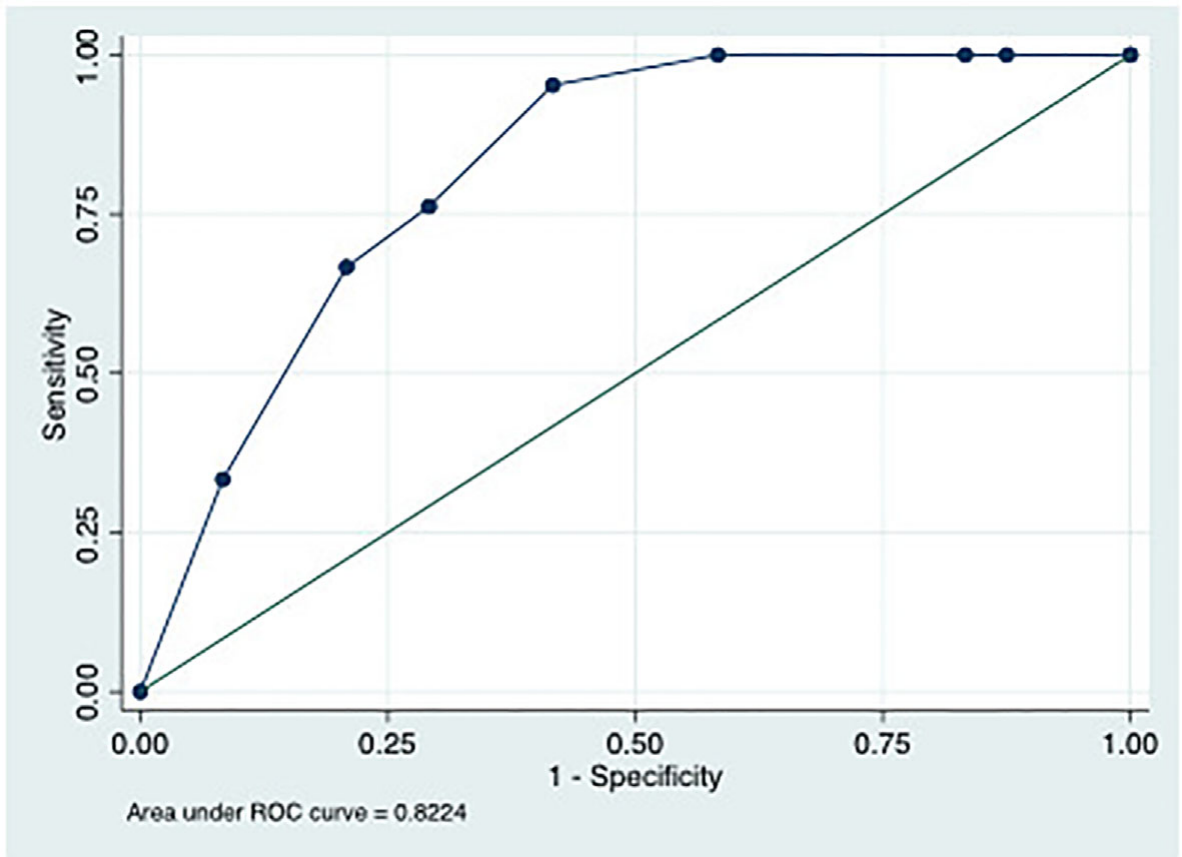
ROC curve of multiple logistic regression model using LPB>20040 ng/mL, EndoCabIgM <97.42 MU/mL and I-FABP pg/mL lower than 689.7 as predictors of ICU admission.

The optimal cut-offs at T0 differentiating ICU from non-ICU patients were 20040 ng/mL (sensitivity 75%, specificity 54%), 97.42 MU/mL (sensitivity 85%, specificity 41%) and 689.7 pg/mL (sensitivity 66.7%, specificity 62.5%) for LBP, EndoCab IgM and I-FABP, respectively. Following simple regression model, the corresponding AUC were 0.67 (0.54-0.80), 0.63 (0.51-0.76) and 0.64 (0.52-0.71) (**Table 2**). LBP>20040 ng/mL and EndoCab IgM <97.42 MU/mL at T0 were associated with ICU admission (OR 5.02, IC 1.29-19.43, p=0.019 and OR 4.28, IC 0.98-18.58, p=0.05, respectively). I-FABP>689.7 pg/mL showed a trend towards protection from ICU admission (OR 0.30, IC 0.90-1.04, p=0.06). Through multivariable analysis, patients with LPB >20040 ng/mL, EndoCab IgM <97.42 MU/mL and I-FABP pg/mL lower than 689.7 were likely to be admitted to ICU (OR 9.97 IC 1.84-53.81, p=0.007, OR 7.70, IC 1.29-45.8, p=0.025, OR 0.21, IC 0.04-1.00, p=0.05, respectively, AUC of the model 0.822) (**Figure 4**).

**Table 2:** Test performance of LBP, EndoCab IgM and I-FABP at T0 for prediction of ICU admission.

Prediction ICU *vs* non ICU	Optimal cut-off	Se	Sp	AUC (CI 95%)
LBP, ng/ml	20040	0.75	0.54	0.67 (0.54-0.80)
EndoCab IgM, MU/mL	97.42	0.85	0.41	0.63 (0.51-0.76)
I-FABP, pg/mL	689.7	0.66	0.62	0.64 (0.52-0.71)

Optimal cut-off for each marker was derived by Youden’s Index. Se, sensitivity; Sp, specificity. LPB, Lipopolysacharide Binding Protein; I-FABP, Intestinal Fatty Acid Binding Protein; T0, sample collected at the moment of COVID-19 diagnosis.

Following Youden’s index, the optimal cut-offs at T0 differentiating survivors from non-survivors were 39750 ng/mL (sensitivity 62.5%, specificity 84.3%), 53.92 MU/mL (sensitivity 63.6%, specificity 67.6%) and 644.7 pg/mL (sensitivity 63.1%, specificity 61%) for LBP, EndoCab IgM and I-FABP, respectively. Following simple regression model, the corresponding AUCs were 0.76 (0.60-0.91), 0.65 (0.48-0.82) and 0.61 (0.48-0.72). However, only LBP>39750 ng/mL was statistically associated with the outcome (OR 10.28, IC95% 2.14-49.23, p=0.004) whereas EndoCab IgM <53.92 MU/mL at T0 showed a trend on association with mortality (OR 3.75, IC95% 0.88-15.18, p=0.07). Following multivariable analysis, only thrombotic events were associated with mortality (**Table 3**).

**Table 3:** Factors associated with in-hospital mortality.

Determinants	Univariable analysis			Multivariable analysis		
	OR	CI95%	p-value	OR	CI95%	p-value
Age	1.03	0.95-1.11	0.416			
Sex	0.86	0.16-4.66	0.86			
BSI	6.25	1.28-30	**0.023**	9.24	0.63-130	0.104
Steroids	3.75	0.60-23.6	0.156			
Thrombotic events	16	2.00-127	**0.009**	32.03	1.83-550	**0.017**
LBP T0>39750 ng/ml	10.28	2.14-49.23	**0.004**	6.51	0.45-92.39	0.166
EndoCab IgM T0 <53.92 MU/mL	3.75	0.88-15.18	0.07			
I-FABP T0>644.7 pg/mL	0.44	0.09-1.63	0.201			

LPB, Lipopolysacharide Binding Protein; I-FABP, Intestinal Fatty Acid Binding Protein; T0, sample collected at the moment of COVID-19 diagnosis. BSI, bloodstream infection. Bold values are statistically significant values.

## Discussion

In the present report, we demonstrated that i) patients with COVID-19 exhibited higher levels of gut leakage and intestinal injury than healthy controls, which were maintained over the course of disease; ii) patients with severe form of COVID-19 (i.e. subjects admitted to ICU) had higher values of MT, but not of ID, than subjects without severe disease, which was, again, maintained over time; iii) patients who developed n-BSI had a high degree of consumption of neutralizing antibodies against LPS endotoxin core antigen over the course of disease, and iv) high degree of MT and low ID are predictors of ICU admission.

The involvement of gut during COVID-19 is not uncommon and is mostly explained by the presence of ACE-2 receptors in the intestinal cells (20, 21). Amongst others, related clinical and laboratory findings include gastrointestinal symptoms in up to one third of patients with COVID-19 (10, 22), the presence of the virus within intestinal cells or in small vessels in the intestine (8) and the persistence of viral RNA in feces (21–23).

However, data regarding the integrity of gut mucosa and the possible intestinal cell damage during the course of the disease are extremely scarce (24–26).

A recent observational study showed that patients with COVID-19 had elevated plasma levels of LBP and CCL-25, but not of I-FABP and, among them, subjects with cardiac involvement exhibited the highest levels of these biomarkers, suggesting a potential gut-heart axis in COVID-19 (24). Likewise, we demonstrated that impairment of gut mucosal barrier occurs during COVID-19 and, to the best of our knowledge, it is herein reported for the first time that this phenomenon is observed especially in severe forms of COVID-19. To this extent, we also showed that the extent of intestinal damage is high in patients with COVID-19 and this represents an additional novelty of the present study, since it is described for the first time. However, patients with severe form of disease had significantly lower level of intestinal injury compared to patients without severe form of infection, possibly suggesting that the main factor contributing to the severity of disease might be represented by the gut leakage itself rather than the intestinal cell damage. In line with this evidence, we found that patients with high degree of MT and low ID (expressed as LPB >20040 ng/mL and EndoCabIgM <97.42 MU/mL and I-FABP pg/mL <689.7, respectively) were likely to be admitted to ICU.

While ACE-2 expression on enterocytes may serve as site for SARS-CoV-2 entrance and predispose to enteric infection leading to degeneration of the gut mucosal integrity, on the other side a dysfunctional gut mucosal barrier facilitates passage of bacteria or their products from the gut to systemic circulation, thus priming a vicious cycle and contributing to disease severity by boosting the systemic inflammation (9, 27).

In fact, the presence of circulating LPS induces toll-like-receptor 4 activation and the production of host response molecules such as LBP and the consumption of neutralizing antibodies against LPS endotoxin core antigen as well as the activation of the inflammatory host defence *via* binding to soluble CD14, which, in turn, initiates the production of several cytokines (9, 28). Furthermore, IL-6 mediated vascular damage may also contribute to increase intestinal permeability, precipitate microbial translocation and perpetuate systemic inflammation (9). Finally, other ‘dangerous molecules’ through the mesenteric lymph and, therefore, not filtered by the liver, can enter directly into the thoracic duct and subsequently into the central circulation causing toxic effects on the pulmonary microvasculature and therefore predisposing to the exasperation of the disease or to the development of different infections.

In our study, high levels of LBP were predictors of in-hospital mortality at univariable analysis, whereas with multivariable investigation only the presence of thrombotic events was an indipendent risk factor for mortality. This is in line with the recent literature, which has clearly demonstrated that venous and arterial thrombotic events are common in COVID-19 and might be therefore responsible for the observed excess of death (29, 30). Beside the increase of pro-inflammatory and pro-thrombotic effects of Angiotensin II as a consequence of the reduction in the expression of ACE-2 (31, 32), an intriguing hypothesis resides also on the presence of circulating levels of LPS and microbial products following gut leakage, which are able to increase the thrombotic state (26). Therefore, further larger studies are warranted to test whether markers of gut leakage are associated with thrombotic events or adverse outcomes in COVID-19.

The development of n-BSI in COVID-19 has been increasingly described (15, 33, 34). However, whether the infection itself or the prolonged hospitalization together with common risk factors for BSI or a combination of the two represents the primary condition for n-BSI is still unknown. Starting from the observation that n-BSI occur frequently after CDI, probably as a consequence of gut mucosal perturbation (18), and taking into consideration the possibility that this phenomenon may be present also in COVID-19, we compared patients with and without BSI and we observed that patients who developed n-BSI had a high degree of consumption of neutralizing antibodies against LPS endotoxin core antigen over the course of disease, expression of higher mucosal perturbation. However, definite conclusions could not be drawn due to the low number of subjects enrolled. Anyway, gut involvement (expressed as increased mucosal permeability) may contribute to enhance the systemic inflammatory response during the course of the disease (9, 35–39).

The present research has several limitations, such as i) the low number of included patients, which also may have hampered the predictive values of MT and ID markers, ii) the relatively low number of HDs, who were matched for sex, age and study period, the latter definitely influencing the sample size, iii) the prevalent descriptive nature of the study and iv) the lack of information regarding the time to obtaining mucosal integrity restoration after disease resolution. Nevertheless, we believe that crucial information on the status of gut mucosa permeability and intestinal cells are obtained by the present study. Should our preliminary data be confirmed in larger studies, the extent of microbial translocation might be considered as a useful tool for identifying patients at higher risk of adverse outcomes.

## Conclusions

We demonstrated that COVID-19 is associated with persistent gut permeability and a certain degree of intestinal damage. The extent of gut mucosal alteration in COVID-19 may represent a useful tool for the early identification of patients at risk of more severe infection and ICU admission. With this in mind, potential therapeutic approaches aiming at restoring the mucosal integrity (i.e. probiotics) should be further investigated. Further investigations are needed to fully elucidate the exact role of microbial translocation and intestinal damage in both pathogenesis and prognosis of COVID-19.

## Data Availability Statement

The original contributions presented in the study are included in the article/**Supplementary Material**. Further inquiries can be directed to the corresponding author.

## Ethics Statement

The studies involving human participants were reviewed and approved by Ethical Committee of Policlinico Umberto I. The patients/participants provided their written informed consent to participate in this study.

## Author Contributions

Study concept and design: AO. Acquisition of data: VM, RA, GC, and CP. Performing experiments: MCM, FT, MA, and DAI. Analysis and interpretation of data: AO. Drafting of the manuscript: AO. Critical revision of the manuscript for important intellectual content: MTM. Gd’E and CP. Statistical analysis: AO. Study supervision: MCM. All authors contributed to the article and approved the submitted version.

## Conflict of Interest

The authors declare that the research was conducted in the absence of any commercial or financial relationships that could be construed as a potential conflict of interest.

## Abbreviations

SARS-CoV-2, Severe Acute Respiratory Syndrome Coronavirus 2; COVID-19, Coronavirus Disease-19; ACE-2, Angiotensin-converting enzyme-2; MT, microbial translocation; ID, intestinal damage; BSI, bloodstream infection; n-BSI, nosocomial bloodstream infection; CDI, *Clostridioides difficile* infection; LPB, Lipopolysacharide Binding Protein; LPS, Lipopolysacharide; I-FABP, Intestinal Fatty Acid Binding Protein; HD, healthy donors; ICU, Intensive Care Unit; IQR, interquartile range.
