# Supplementary material for: Persistent Systemic Microbial Translocation and Intestinal Damage During Coronavirus Disease-19
Source: Front Immunol. 2021 Jul 14;12:708149. doi: 10.3389/fimmu.2021.708149 (PMC8316921; doi:10.3389/fimmu.2021.708149)
Supplement: Supplementary file 1 [file Table_1.docx]

| **Characteristics** | **Intensive Care Unit admission**  **(n=21)** | **No Intensive Care Unit admission**  **(n=24)** | ***p value*** |
| --- | --- | --- | --- |
| Age, years, median (IQR) | 68 (58-76.7) | 62 (52-68) | n.s. |
| Sex (M/F), n | 11/9 | 14/10 | n.s. |
| Duration of symptoms, days, median (IQR) | 3.1 (2.1-10.7) | 7.6 (2.1- 15.5) | n.s. |
| **Blood analyses, median (IQR)** |  |  |  |
| White Blood Cells, x10^6^/L | 8055 (4830- 9383) | 5670 (5160-7330) | n.s. |
| Neutrophils, x10^6^/L | 7255 (4135-8625) | 4240 (3420- 6030) | n.s. |
| Lymphocytes, x10^6^/L | 705 (497.5-1073) | 1000 (610-1390) | 0.07 |
| Monocytes, x10^6^/L | 360 (190-510) | 380 (250-510) | 0.54 |
| Platelets, x10^9^/L | 217 (171-280) | 189 (176-248) | n.s. |
| Albumin, g/L | **3.4 (3.1-3.7)** | **4.1 (3.4-4.4)** | **0.003** |
| D-Dimer, µg/L | **2115 (568- 4407)** | **492 (330.5- 925.3)** | **0.004** |
| Fibrinogen, mg/dL | 560 (521.3- 593) | 556 (393-560) | n.s. |
| PaO2/FiO2 ratio | **246.5 (178.3- 265.3)** | **359.5 (302.5-390.5)** | **0.0006** |
| **Comorbidities, n (%)** |  |  |  |
| Smoke | 2 (9.5) | 1 (4.2) | n.s. |
| Diabetes mellitus | 4 (19.1) | 3 (12.5) | n.s. |
| Heart failure | **4 (19.1)** | **0** | **0.04** |
| Vasculopathy | 4 (19.1) | 6 (25) | n.s. |
| Cerebrovascular events | 2 (9.5) | 2 (8.3) | n.s. |
| Asthma | 0 | 0 | n.s. |
| Chronic Obstructive Pulmonary Disease | 3 (14.3) | 1 (4.2) | n.s. |
| AIDS | 0 | 0 | n.s. |
| **Symptoms, n (%)** |  |  |  |
| Fever | 15 (71.4) | 18 (75) | n.s. |
| Cough | 4 (19.1) | 10 (41.7) | n.s. |
| Dispnoea | 11 (52.4) | 9 (37.5) | n.s. |
| Diarrhea | 1 (4.8) | 3 (12.5) | n.s. |
| Headache | 1 (4.8) | 1 (4.2) | n.s. |
| Fatigue | 0 | 2 (8.3) | n.s. |
| **Therapy, n (%)** |  |  |  |
| Hydroxychloroquine | 16 (76.2) | 17 (75) | n.s. |
| Azithromycin | 6 (28.6) | 13 (54.2) | n.s. |
| Protease Inhibitors | 1 (4.8) | 6 (25) | n.s. |
| Tocilizumab | 10 (47.6) | 11 (45.8) | n.s. |
| Steroids | 8 (38.1) | 7 (29.2) | n.s. |
| Enoxaparin | 11 (52.4) | 13 (54.2) | n.s. |
| Teicoplanin | **8 (38.1)** | **1 (4.2)** | **0.0073** |
| **Outcomes, n (%)** |  |  |  |
| Bloodstream Infections | **9 (42.8)** | **0 (0)** | **0.0003** |
| Trombotic events | 4 (19.1) | 2 (8.3) | n.s. |
| Death | **10 (47.6)** | **0** | **0.0001** |

**Supplementary Table1**. Characteristics of patients with COVID-19 admitted to Intensive care Unit (n=21) or not.(n=24) AIDS: Acquired Immune Deficiency Syndrome. Ns: not significant.
