# Supplementary material for: Persistent Systemic Microbial Translocation and Intestinal Damage During Coronavirus Disease-19
Source: Front Immunol. 2021 Jul 14;12:708149. doi: 10.3389/fimmu.2021.708149 (PMC8316921; doi:10.3389/fimmu.2021.708149)
Supplement: Supplementary file 2 [file Table_2.docx]

| **Characteristics** | **Subjects with bloodstream infection (n=9)** | **Subjects without bloodstream infection (n=36)** | ***p value*** |
| --- | --- | --- | --- |
| Age, years, median (IQR) | **73 (67-83)** | **62 (54.5-68.75)** | **0.04** |
| Sex (M/F), n | 6/3 | 21/15 | n.s. |
| Duration of symptoms, days,  median (IQR) | 4.5 (2.58-9.6) | 4.4 (2.9-10.6) | n.s. |
| **Blood analyses, median (IQR)** |  |  |  |
| White Blood Cells, x10^6^/L | 9090 (6740-11400) | 5775 (48.75-77.48) | n.s. |
| Neutrophils, x10^6^/L | 7630 (4720-9960) | 4340 (3430-6858) | n.s. |
| Lymphocytes, x10^6^/L | 490 (350-1140) | 825 (617.5-1385) | n.s. |
| Monocytes, x10^6^/L | 360 (290-550) | 360 (240-510) | n.s. |
| Platelets, x10^9^/L | 232 (136-286) | 193 (176-241) | n.s. |
| Albumin, g/L | **3.3 (2.8-3.7)** | **3.8 (3.4- 4.3)** | **0.02** |
| D-Dimer, µg/L | **4160 (1260-4610)** | **515 (346.3-1331)** | **0.001** |
| Fibrinogen, mg/dL | 556 (467-659) | 560 (427.5-560) | n.s. |
| PaO2/FiO2 ratio | **182.5 (153-251)** | **326.5 (271.8-384.8)** | **0.0016** |
| **Comorbidities, n (%)** |  |  |  |
| Smoke | 1 (11.1) | 2 (5.5) | n.s. |
| Diabetes mellitus | 3 (33.3) | 4 (11.1) | n.s. |
| Heart failure | **3 (33.3)** | **1 (2.8)** | **0.02** |
| Vasculopathy | 3 (33.3) | 7 (19.4) | n.s. |
| Cerebrovascular events | 2 (22.2) | 2 (5.6) | n.s. |
| Asthma | 0 (0) | 0 (0) | n.s. |
| Chronic Obstructive Pulmonary Disease | 2 (22.2) | 2 (5.6) | n.s. |
| AIDS | 0 (0) | 0 (0) | n.s. |
| **Symptoms, n (%)** |  |  |  |
| Fever | 7 (77.8) | 26 (72.2) | n.s. |
| Cough | 2 (22.2) | 12 (33.3) | n.s. |
| Dispnoea | 5 (55.6) | 15 (41.7) | n.s. |
| Diarrhea | 0 (0) | 4 (11.1) | n.s. |
| Headache | 0 (0) | 2 (5.6) | n.s. |
| Fatigue | 0 (0) | 2 (5.6) | n.s. |
| **Therapy, n (%)** |  |  |  |
| Hydroxychloroquine | 6 (66.7) | 27 (75) | n.s. |
| Azithromycin | 3 (33.3) | 16 (44.4) | n.s. |
| Protease Inhibitors | 1 (11.1) | 6 (16.7) | n.s. |
| Tocilizumab | 2 (22.2) | 19 (52.8) | n.s. |
| Steroids | 4 (44.4) | 11 (30.6) | n.s. |
| Enoxaparin | 5 (55.6) | 19 (52.8) | n.s. |
| Teicoplanin | 3 (33.3) | 6 (16.7) | n.s. |
| **Outcomes, n (%)** |  |  |  |
| Intensive Care Unit admission | 9 (100) | 12 (33.3) | **0.0003** |
| Trombotic events | 2 (22.2) | 4 (11.1) | n.s. |
| Death | 5 (55.6) | 5 (13.9) | **0.02** |

**Supplementary Table2**. Characteristics of patients with COVID-19 with (n=9) and without (n=36) bloodstream infection AIDS: Acquired Immune Deficiency Syndrome. Ns: not significant.
